# Effects of physical exercise on biomarkers of oxidative stress in healthy subjects: A meta-analysis of randomized controlled trials

**DOI:** 10.1515/biol-2022-0668

**Published:** 2023-08-08

**Authors:** Yahai Wang, Donglin Luo, Haichao Jiang, Yu Song, Zhiqiang Wang, Lin Shao, Yuxiao Liu

**Affiliations:** College of Arts and Physical Education, Nanchang Normal College of Applied Technology, Nanchang, 330108, Jiangxi Province, China; Faculty of Health Service, Naval Medical University, Shanghai, 200433, China; Military and Political Basic Teaching and Research Office, Army Military Transportation University, Zhenjiang, 212003, Jiangsu Province, China; School of Nursing, Naval Medical University, Shanghai, 200433, China

**Keywords:** physical exercise, oxidative stress, meta-analysis.

## Abstract

This meta-analysis investigated the effect of physical exercise (PE) on the levels of oxidative biomarkers in randomized controlled trials (RCTs) involving healthy subjects. We searched five databases for articles until May 1, 2023. A random-effect meta-analysis, subgroup analysis, meta-regressions as well as trim and fill method were conducted using STATA 11.0, involving ten articles. According to the results of the meta-analysis, PE had no significant effect on superoxide dismutase (SOD), glutathione peroxidase, and catalase levels. PE induced significant increase in total antioxidant status (standardized mean difference [SMD] 1.53, 95% CI 0.73–2.32), and PE could significantly reduce the level of malondialdehyde (MDA) (SMD −1.11, 95% CI −2.15 to −0.06). Sensitivity analyses and subgroup analyses showed that male participants, body mass index (BMI) <25, exercise duration between 1 and 12 weeks, resistance exercise or multicomponent exercise, and exercise of low or moderate intensity were associated with a significant PE-induced decrease in MDA concentrations. Meta-regression analysis identified the age of the participants as a confounder of the effect of PE on SOD levels. The older age of the subjects was associated in a gradient fashion with incident SOD levels. Further RCTs are required to investigate the optimal PE protocol for people of different ages and BMI as well as the effect of PE on oxidative stress.

## 1 Introduction

Globally, roughly 27.5% of adults [1] and 81% of adolescents [2] are physically inactive, which increases 6–10% risk of chronic diseases, premature death [3], and the risk of dementia [4]. Physical exercise (PE) is a non-pharmaceutical method for preventing diseases in which the antioxidant defense plays a key role [5,6]. Additionally, PE could reduce insulin resistance [7,8] which is related to the inhibition of inflammation [9,10] and oxidative stress [11]. Therefore, it is essential to explore the influence of PE on oxidative stress, which is a key pathogenic element in the onset and progression of numerous chronic diseases [12,13].

Oxidative stress is associated with the progression of many non-communicable diseases [13,14] and is characterized by an increase in reactive oxygen species (ROS) and reactive nitrogen species. PE can temporarily induce the production of ROS [15], which results in a compensatory increase in antioxidant levels. This contributes to exercise-induced adaptation of skeletal muscle *in vivo* [16], eventually producing positive or harmful effects according to the activated oxidoreductive status [17,18]. The effects of PE on oxidative stress have been a source of contentious debate, mainly because the changes in oxidative metabolism vary greatly with the exercise protocols and populations, making it difficult to reach a consensus.

The primary objective of this meta-analysis is to investigate the association between exercise intervention and oxidative stress in order to make recommendations for future exercise interventions.

## 2 Materials and methods

### 2.1 Literature search

This meta-analysis followed the Preferred Reporting Items for Systematic Reviews and Meta-Analyses (PRISMA) 2020 guidelines [19]. On the basis of the Population, Intervention, Comparator, Outcome, and Study design framework, articles published between 1980 and May 1, 2023 were retrieved from PubMed, Web of Science, Cochrane Library, Scopus, and Embase. The following search strategy was used: (“Exercise” OR “Physical Exercise” OR “Exercise Therapy”) AND (“Oxidative Stress” OR “Antioxidants”) AND “randomized controlled trial.” Detailed search strategies are shown in Table S1.

### 2.2 Study selection

Two researchers (Y.H.W. and D.L.L.) independently screened titles and abstracts, followed by the full-text, to determine eligibility. The third researcher (H.C.J.) arbitrated any disagreements so that a consensus could be reached. In addition, we manually searched for references to include publications and pertinent review articles. The study includes research and review publications that met the following inclusion criteria: (1) randomized controlled trials (RCTs) that are parallel or crossover; (2) the volunteers were healthy individuals; (3) the intervention of interest was any intensity, frequency, or duration of PE; PE versus non-exercise control or PE plus other intervention versus other intervention only were compared; (4) the outcomes of focus were plasma or serum biomarkers for oxidative stress; and (5) articles published after 1980. The exclusion criteria included: (1) studies involving sick individuals and (2) studies lacking baseline data or data on the final assessment of outcomes in both intervention groups and comparators used to calculate mean changes of treatment ± standard deviation (SD).

### 2.3 Data extraction and quality assessment

Two trained researchers (Y.H.W. and D.L.L.) performed independent data extraction and quality evaluation. The retrieved data comprised the surname of the first author, publication year, study design, study location, sample size, participants’ age and gender, participants’ baseline body mass index (BMI), PE intervention (duration, kind, intensity, frequency), and the results of reported biomarkers. Two or more PE intervention strata (e.g., different forms, intensities, frequencies, or durations of PE) were analyzed as distinct trials. This review included all sorts of PE (aerobic exercise, resistance exercise, and multicomponent exercise). In accordance with American College of Sports Medicine recommendations, the intensity of PE was categorized by maximal heart rate (HRmax), maximal oxygen uptake (VO_2_peak), and maximum repetitions (RM) [20]. The methodological quality and risk of bias of each included study were evaluated using the PEDro scale [21]. The Grading of Recommendation, Assessment, Development, and Evaluation (GRADE) system was used to assess the evidence level of each outcome [22]. According to the GRADE standards, research design determines the baseline quality of the evidence (RCTs were previously considered as high quality), but other factors may diminish (e.g., unexplained heterogeneity) or increase (e.g., a significant magnitude of effect) the studies’ quality [22]. Disagreements were resolved via dialogue with the third reviewer (H.C.J.).

### 2.4 Statistical analysis

There were differences among the included studies in study participants, PE protocols employed, and the measurement of oxidative biomarkers. Thus, the random effects model was used to pool estimates of net changes (changes of intervention group minus changes of control group) in the concentrations of biomarkers, and the results were presented as standardized mean difference (SMD) with 95% confidence intervals (CIs). According to the technique proposed by Wan et al. [23], advanced data extraction was employed for studies that did not directly give either the baseline or final mean and SD of outcomes.

Initially, a primary meta-analysis was performed to determine the overall impact of PE on each oxidative biomarker. On the basis of the quality of the studies, sensitivity analyses were conducted to determine the robustness of the initial meta-analysis’ conclusions. Sources of potential heterogeneity were identified by carrying out subgroup analyses based on region, gender, age of participants, baseline BMI of participants, duration of intervention, type of PE, and intensity of PE (only conducted if more than six trials reported the same outcomes). Using meta-regression analysis, differences between groups and causes of heterogeneity were analyzed. Further meta-regression analyses were conducted to investigate potential moderators and examine the association between moderators and outcomes, with *p*-value <0.1 being considered as statistically significant. The heterogeneity among studies was tested using Cochrane’s *Q* test and quantified using *I* ^2^-statistic [24]. *I* ^2^ > 50% and *p* < 0.10 for the *Q* test show the presence of heterogeneity. Begg’s and Egger’s regression tests and funnel plots were used to evaluate publication bias, with a *p* < 0.10 indicating the presence of bias [25]. If publication bias was detected, the trim and fill approach was employed to eliminate very small studies and recalculate the pooled effect until the funnel plot was symmetrical [26]. All analyses were performed using STATA version 11.0 (Stata Corp, College Station, TX, USA), with double data input to avoid input errors. *p* < 0.05 was deemed as statistically significant unless specified elsewhere.

## 3 Results

### 3.1 Flow of study selection

**Figure 1:**
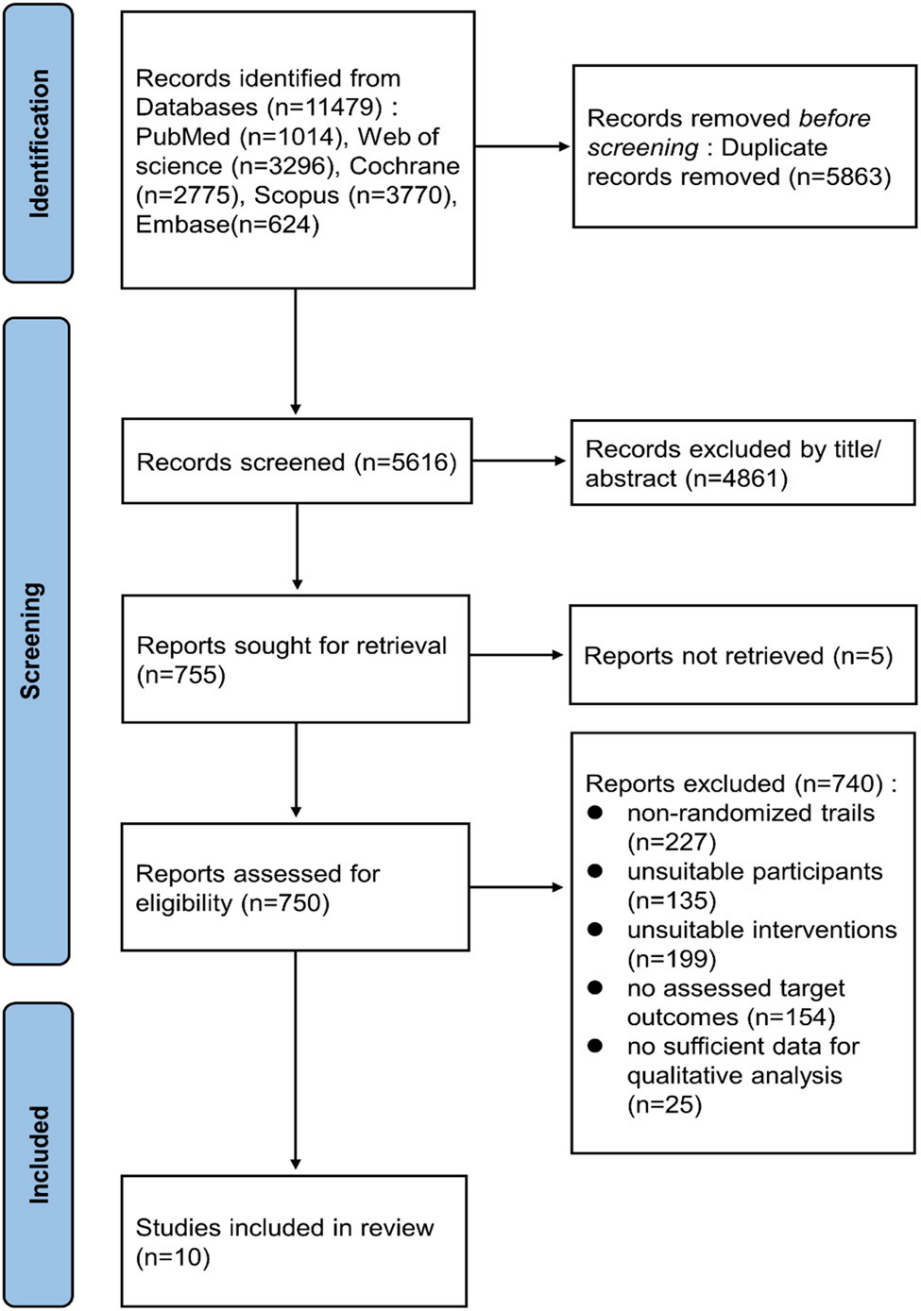
Flowchart showing the procedure used for study selection.

The flowchart depicting the study selection procedure is shown in Figure 1. Initially, 11,479 publications were retrieved, but after excluding the duplicates and screening the titles and abstracts, only 750 articles were left for full-text review. A further 740 articles were eliminated for the following reasons: 227 articles did not involve RCT design, participants of 135 studies were not healthy, 199 articles had improper intervention or control, assessable target outcomes were not reported in 154 articles, and 25 articles lacked sufficient data for quantitative analysis. Finally, ten studies [27–36] involving 458 participants were included in the final meta-analysis.

### 3.2 Qualities of included studies and outcome measure evidence by GRADE system

The quality of the methodology of ten studies were rated as good quality (6–8 scores) mainly due to lack of blinding according to the PEDro scale (Table 1). According to the GRADE system, the quality of evidence for malondialdehyde (MDA) and total antioxidant status (TAS) was moderate, while the quality of evidence for superoxide dismutase (SOD), glutathione peroxidase (GPx), and catalase (CAT) was low (Table 2).

**Table 1:** Characteristics of included studies in this meta-analysis (ten studies)

Study	Design	Country	Subject	Comparators	Interventions	Duration of intervention	Method of measuring/biomarkers outcomes	Quality^a^
Alghadir, 2016	RP	Saudi Arabia	Healthy subjects (***N*** **= 100**)	**Control (** ***N*** **= 50):** Not described.	**Aerobic exercise (** ***N*** **= 50):** stretching exercises and walking (5–10 min); treadmill, bicycle, and stair training (45–60 min).	24 wk	ELISA/TAS, MDA	Good
					*Intensity:* 30–45% of VO_2_max			
					*Frequency:* 3 times/wk			
Azizbeigi, 2015	RP	Iran	Male physical education students (***N*** **= 30**)	**Control (** ***N*** **= 10):** Not described.	**Resistance exercise (** ***N*** **= 20):** upper body and lower body exercises: chest press, lat pull down, leg extension and flexion, biceps and triceps curls, squats, and sit-ups.	8 wk	Not described/SOD, GPx, MDA	Good
					*Intensity:* 65–70% (moderate intensity group, *N* = 10)/85–90% (high intensity group, *N* = 10) of 1RM.			
					*Frequency:* 3 times/wk			
Alikhani, 2019	RP	Iran	Young and older adult women (***N*** **= 24**)	**Control (** ***N*** **= 24):** No exercise.	**Resistance exercise (** ***N*** **= 24):** 10 min to warm-up, 45 min for the main training phase and 5 min for cool-down in each session.		ELISA/MDA, TAS	Good
					*Intensity:* 75% of 1RM.			
					*Frequency:* 3 times/wk			
Bunpo, 2016	RP	Thailand	Healthy untrained adult (***N*** **= 26**)	**Control (** ***N*** **= 16):** Sedentary	**Aerobic exercise (** ***N*** **= 10):** outdoor running (30 min).	12 wk	Thiobarbituric acid reactive substances/TAS, SOD, MDA	Good
					*Intensity:* 65–75% of HRmax			
					*Frequency:* 3 times/wk			
Dani, 2021	RP	Brazil	Healthy elderly women (***N*** **= 10**)	**Control (** ***N*** **= 9):** Sedentary	**Multicomponent exercise (** ***N*** **= 31):** 10 min warm-up and 40 min of strengthening, balance, functional capacity, motor coordination and cognition exercises, resistance exercises for the upper sessions, squatting associated with the resistance motor task, among other variations. 10 min stretching exercises.		Spectrometer/SOD, CAT	Good
					*Intensity:*/			
					*Frequency:* 2 times/wk			
Fatouros, 2004	RP	Greece	Healthy older men (***N*** **= 19**)	**Control (** ***N*** **= 8):** Non-exercising.	**Aerobic exercise (** ***N*** **= 11):** warm-up (3–5 min); treadmill walking/jogging (12–42 min).	16 wk	Spectrometer/TAS, MDA, GPx	Good
					*Intensity:* 50–80% of HRmax			
					*Frequency:* 3 sessions/wk			
Garten, 2019	RC	USA	Healthy males (***N*** **= 20**)	**Control (** ***N*** **= 10):** Remained sitting position for 3 h with legs stationary.	**Aerobic exercise (** ***N*** **= 10):** sit session as control; warm-up (10 min); high-intensity interval aerobic cycling exercise (28 min); sit session as control (3 h)	3 h	Not described/MDA, SOD	Good
					*Intensity:* 85–95% HRmax			
Goon, 2009	RP	Malaysia	Healthy volunteers (***N*** **= 84**)	**Control (** ***N*** **= 44):** Sedentary volunteers.	**Aerobic exercise (** ***N*** **= 40):** Tai-Chi (1 h).	24 wk, 48 wk, 24 wk	Spectrometer/MDA, GPx, SOD, CAT	Good
					*Intensity:* not mentioned			
					*Frequency:* 2 times/wk			
Rosado-perez, 2012	RP	México	Elderly subjects (***N*** **= 55**)	**Control (** ***N*** **= 23):** No intervention.	**Aerobic exercise (** ***N*** **= 32):** daily training in Tai Chi (50 min).	24 wk	Other/GPx, SOD, TAS	Good
					*Intensity:* not mentioned			
					*Frequency:* 3 h-long sessions/wk			
Soares, 2015	RP	Portugal	Healthy Caucasian men (***N*** **= 57**)	**Control (** ***N*** **= 26):** Did not undergo any physical activity.	**Multicomponent exercise (** ***N*** **= 31):** aerobic exercise (walking, running, biking, rowing, and elliptical) (25–30 min), strength exercise (bench press, leg press, leg curl, leg extension, latissimus, abdominals, and arm flexion) (30–35 min), stretching and cool down (5–10 min).	16 wk	Spectrometer/MDA, TAS	Good
					*Intensity:* 75% of HR reserve (aerobic exercise); 75% of 1RM (strength exercise)			
					*Frequency:* 3 sessions/wk			

^a^Better methodological quality is indicated by a higher PEDro score (9–10: excellent; 6–8: good; 4–5: fair; <4: poor); significant *p*-values (*p* < 0.05) are highlighted in bold prints.

Abbreviations: CAT, catalase; Db, double blind; kcal, kilocalories; km, kilometer; GPx, glutathione peroxidase; HRmax, maximal heart rate; MDA, malondialdehyde; min, minute; RC, randomized crossover; RM, repetition maximum; RP, randomized-parallel; s, second; SB, single-blinded; SOD, superoxide dismutase; TAS, total antioxidant status; VO_2_max, maximal oxygen uptake; wk, week.

**Table 2:** Grades of recommendation, assessment, development, and evaluation (GRADE) quality of evidence

Outcome	Risk of bias	Inconsistency	Indirectness	Imprecision	Publication bias	Effect size	Plausible residual confounding	Dose–response gradient	GRADE rating
MDA	0	−1^a^	0	0	0	0	0	0	Moderate
SOD	0	−1^a^	0	−1^b^	0	0	0	0	Low
TAS	0	−1^a^	0	0	0	0	0	0	Moderate
GPx	0	−1^a^	0	−1^b^	0	0	0	0	Low
CAT	0	−1^a^	0	−1^b^	0	0	0	0	Low

^a^Significant and unexplained variability exists in the primary analysis. ^b^Effect size overlaps 0 in the primary analysis. ^c^ *p* < 0.1 on Begg’s or Egger’s regression test. Abbreviations: CAT, catalase; GPx, glutathione peroxidase; MDA, malondialdehyde; SOD, superoxide dismutase; TAS, total antioxidant status.

### 3.3 Characteristics of included studies

Characteristics of studies included in this meta-analysis are shown in Table 1. The final sample consisted of 458 participants. Sample sizes ranged from 19 to 100 participants, with a median size of 42.5. Eight studies were carried out in different countries including eight countries each with a single study, and two studies in Iran. Different types of PE (aerobic exercise: six interventions; resistance exercise: three interventions; multicomponent exercise: three intervention) were evaluated in the studies.

### 3.4 Effect of PE on pro-oxidant biomarkers

**Figure 2:**
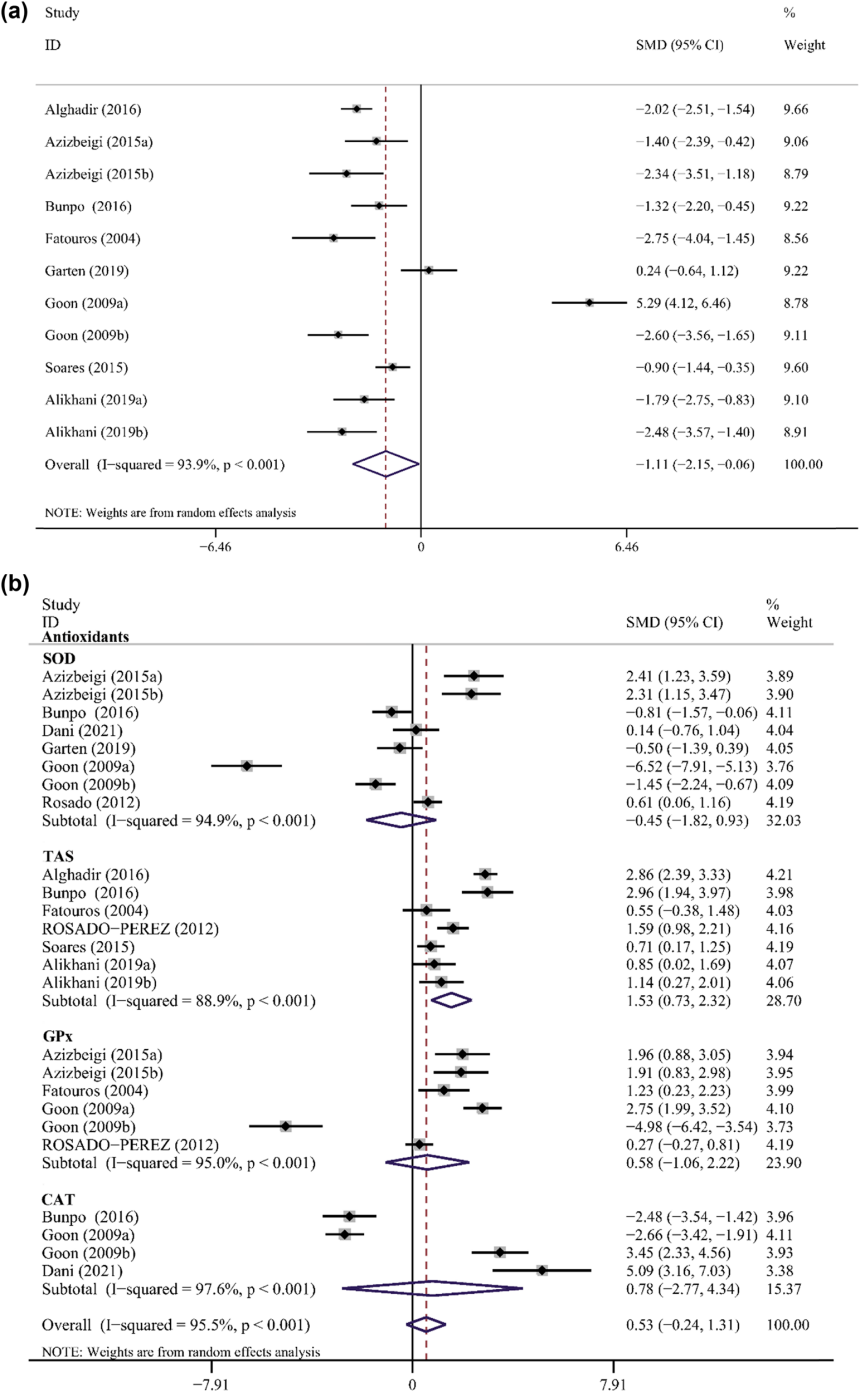
Forest plot of the effect of PE on MDA levels (a) and antioxidants (b).

Assessment of MDA levels was reported in eight articles including 11 interventions [27,29,30,32–34,36]. The primary meta-analysis showed that PE could significantly reduce the level of MDA (SMD −1.11, 95% CI −2.15 to −0.06). In addition, a high heterogeneity was observed among the studies (*p* < 0.001, *I* ^2^ = 93.9%) (Table 3 and Figure 2a). Sensitivity analyses and subgroup analyses showed that male participants, BMI < 25, exercise duration between 1 and 12 weeks, resistance exercise or multicomponent exercise, and exercise of low or moderate intensity were associated with significant PE-induced decrease in MDA concentrations (Table 3). No significant publication bias was found (Table 3). As shown in Table 3, meta-regression analysis did not identify any potential confounders.

**Table 3:** Results of sensitivity analysis, subgroup analysis, and publication bias stratified by study characteristics

Outcomes	Interventions	SMD (95% CI)	*P* ^1^	Heterogeneity		*P* ^3^	*P* ^4^	
				*I* ^2^ (%)	*P* ^2^		Begg’s value	Egger’s value
Pro-oxidant	Interventions	Net change (95% CI)	*P* ^1^	*I* ^2^ (%)	*P* ^2^	*P* ^3^	*P* ^4^	
							Begg’s value	Egger’s value
**MDA**								
**Overall**	11	−1.11 (−2.15, −0.06)	**0.038**	93.9	**<0.001**		1.000	0.227
**Region**						0.635		
Conducted in Asia only	7	−0.92 (−2.80, 0.87)	0.305	95.7	**<0.001**		0.764	0.288
**Gender**						−		
Male only	5	−1.34 (−2.27, −0.41)	**0.005**	80.4	**<0.001**		−	−
**Age**						0.624		
<45 years	5	−1.29 (−2.14, −0.43)	**0.003**	74.5	**0.003**		−	−
45–60 years	3	0.57 (−3.21, 4.36)	0.767	98.2	**<0.001**		−	−
**≥**60 years	3	−2.17 (−2.59, −1.75)	**<0.001**	0.0	0.490		−	−
**Baseline BMI**						0.223		
<25	3	−1.96 (−2.36, −1.55)	**<0.001**	0.0	0.429		−	−
>25	5	−0.51 (−2.81, 1.78)	0.660	96.9	**<0.001**		−	−
**Duration**						0.974		
<1 day	1	0.24 (−0.64, 1.12)	0.580	−	−		−	−
1–12 weeks	5	−1.79 (−2.24, 1.34)	**<0.001**	2.9	0.390		−	−
>12 weeks	5	−0.62 (−2.68, 1.44)	0.551	97.2	**<0.001**		−	−
**Type of exercise**						0.720		
Aerobic exercise	6	−0.54 (−2.50, 1.41)	0.587	96.7	**< 0.001**		0.452	0.249
Resistance exercise	4	−1.95 (−2.47, −1.43)	**<0.001**	0.0	0.450		−	−
Multicomponent exercise	1	−0.90 (−1.44, −0.35)	**0.001**	−	−		−	−
**Intensity of exercise**						0.385		
Low	1	−2.02 (−2.51, −1.54)	**<0.001**	−	−		−	−
moderate	6	−1.65 (−2.22, −1.07)	**<0.001**	57.7	**0.037**		−	−
high	2	−1.02 (−3.55, 1.51)	0.430	91.7	**0.001**		−	−

Antioxidant	Interventions	Net change (95% CI)	*P* ^1^	*I* ^2^ (%)	*P* ^2^	*P* ^3^	*P* ^4^	
							Begg’s value	Egger’s value
**SOD**								
**Overall**	8	−0.45 (−1.82, 0.93)	0.523	94.9	**<0.001**		0.711	0.625
**Region**						0.765		
Conducted in Asia only	5	−0.80 (−3.28, 1.69)	0.530	96.8	**<0.001**		−	−
**Age**						**0.080**		
<45 years	4	0.80 (−0.86, 2.47)	0.344	91.5	**<0.001**		−	−
45–60 years	2	−3.95 (−8.92, 1.02)	0.119	97.4	**<0.001**		−	−
**Baseline BMI**						0.123		
<25	2	2.36 (1.53, 3.18)	**<0.001**	0	0.908		−	−
>25	4	−2.77 (−5.70, 0.16)	0.064	96.2	**<0.001**		−	−
**Duration**						**0.037**		
<1 day	1	−0.50 (−1.39, 0.39)	0.272	−	−		−	−
1∼12 weeks	5	0.86 (−0.22, 1.94)	0.118	87.4	**<0.001**		−	−
>12 weeks	2	−3.95 (−8.92, 1.02)	0.119	97.4	**<0.001**		−	−
**Type of exercise**						0.108		
Aerobic exercise	5	−1.65 (−3.38, 0.08)	0.062	95.7	**<0.001**		−	−
Resistance exercise	2	2.36 (1.53, 3.18)	**<0.001**	0	0.908		−	−
**Intensity of exercise**						0.849		
Moderate	3	0.53 (−1.19, 2.24)	0.547	90.2	**<0.001**		−	−
High	2	0.88 (−1.87, 3.63)	0.531	93.0	**<0.001**		−	−
**TAS**	7	1.53 (0.73, 2.32)	**<0.001**	88.9	**<0.001**		−	−
**GPx**	6	0.58 (−1.06, 2.22)	0.486	95.0	**<0.001**		1.000	0.508
**CAT**	4	0.78 (−2.77, 4.34)	0.665	97.6	**<0.001**		−	−

*P* ^1^ value for net change; *P* ^2^ value for heterogeneity in the subgroup; *P* ^3^ value for heterogeneity between groups with meta-regression, analyzed as categorical variables; *P* ^4^ value for publication bias (conducted only when trials >5); significant *p*-values (*p* < 0.05) are highlighted in bold prints.

Abbreviations: BMI, body mass index; CAT, catalase; CI, confidence interval; GPx, glutathione peroxidase; MDA, malondialdehyde; RC, randomized crossover; RP, randomized-parallel; SMD, standardized mean difference; SOD, superoxide dismutase; TAS, total antioxidant status.

### 3.5 Effect of PE on antioxidant biomarkers

SOD levels were examined in six studies [29,30,31,33–35] involving eight interventions. The results of the meta-analysis demonstrated that PE had no effect on SOD levels (SMD −0.45, 95% CI −1.82 to 0.93). There was also evidence of high heterogeneity among the studies (*p* < 0.001, *I* ^2^ = 94.9%) (Table 3 and Figure 2b). Significant improvements were only observed in those with baseline BMI < 25, but not in those with baseline BMI > 25. Similarly, significant improvements were only observed in those taking resistance exercise, but not in those taking aerobic exercise. Results of Begg’s and Egger’s tests did not reveal any publication bias (Table 3). Meta-regression analysis identified the age of the participants as a confounder of the effect of PE on SOD levels, with adjusted *R* ^2^ of 53.56%. Older age of subjects was associated in a gradient fashion with incident SOD levels (Table 4). TAS was assessed in six studies [27,28,30,32,35,36]. Results of meta-analysis revealed that PE significantly increased TAS levels (SMD 1.53, 95% CI 0.73–2.32), with a high heterogeneity among the studies (*p* < 0.001, *I* ^2^ = 88.9%) (Table 3 and Figure 2b). No significant effect was found on the levels of GPx (assesses in four studies including five trials) [32,34,35] and CAT (assessed in two studies including four trials) [30,31,34] levels (Table 3 and Figure 2b).

**Table 4:** Meta-regression analysis of potential moderators

Outcome	Covariate	Interventions (*n*)	Coefficient	95% CI	*P*	Covariate adjusted *R* ^2^
MDA	Age^2^	11	−0.005	−0.110 to 0.099	0.907	−
	Baseline BMI^2^	8	0.397	−1.059 to 1.853	0.515	−
SOD	Age^2^	7	−0.170	−0.356 to 0.166	**0.065**	53.56%

^1^Analyzed as continuous variables; ^2^analyzed as categorical variables; significant *p*-values (*p* < 0.05) are highlighted in bold prints.

Abbreviations: BMI, body mass index; CI, confidence interval; MDA, malondialdehyde; SOD, superoxide dismutase.

### 3.6 Publication bias

As shown in Table 3, based on Begg’s and Egger’s tests, there was no publication bias in all results of this meta-analysis.

## 4 Discussion

To the best of our knowledge, this is the first meta-analysis from ten RCTs to estimate the effect of PE on individual biomarkers of oxidative stress among healthy subjects. Overall, our results revealed that PE induced an increase in the TAS level, and reduced the level of MDA. Male participants, BMI < 25, 1–12 weeks of resistance or multicomponent exercise, and low or moderate intensity exercise were all linked to a significant drop in MDA levels caused by PE.

There was a moderate level of evidence in this study that showed that PE induced an increase in the antioxidant capacity of cells by increasing TAS levels. Furthermore, PE significantly reduced MDA levels, which is a pro-oxidant indicator. Our results are consistent with previous meta-analysis involving disease populations [37], which also revealed that any type of PE could increase the level of antioxidants and decrease the levels of pro-oxidants. Oxidative stress is caused by reduced antioxidant capacity resulting from low levels of antioxidants and reduced activity of antioxidant enzymes with or without the production of ROS [38]. Davies et al. [39] first discovered that contracted skeletal muscles produced ROS, with the production site widely assumed as mitochondria [40]. Subsequent studies demonstrated that this effect induced beneficial adaptations to oxidative stress by mediating the activation of the antioxidant system and the repair system of oxidative damage [41,42]. In general, oxidative stress is quantified by measuring the levels of pro-oxidants and antioxidants.

Our results also revealed that PE had no significant effect on levels of SOD, GPx, and CAT. Consistent with our results, a meta-analysis of animal studies showed less than 8 weeks of PE did not affect the levels of SOD, GPx, and CAT [43]. However, they also found that after more than 8 weeks of PE, the levels of SOD and CAT increased, but no significant effect was observed in the GPx level [43]. These results indicate that antioxidant enzyme expression has an adaptive response to exercise, which may be regulated by the dose of PE. Interestingly, we found that age was negatively correlated with PE-induced elevations of SOD levels. Aging is associated with increased ROS production in the skeletal muscle due to abnormal regulation of redox balance, which leads to oxidative damage [41]. At the same time, the sedentary habits and increased risk factors that develop with aging also lead to the decline in antioxidant defense ability. Consequently, the oxidative damage caused by aging may mask the effect of exercise on the circulating levels of SOD. In addition, although the primary results showed a lack of PE-induced effect on SOD levels, subgroup analysis stratified by demographic characteristics and exercise protocols showed significant effects of exercise on SOD levels. However, these results should be interpreted with caution due to the small number of studies and the unexplained heterogeneity across studies.

There are several limitations in this study. First, the included trials lacked blinding, which may have added bias. Due to the considerable heterogeneity identified among research, it is necessary to interpret our study’s findings with caution. Different experimental designs, particularly study samples, interventions, and different outcome measures, may have contributed to the substantial heterogeneity among the research. Although we addressed the cause of heterogeneity by subgroup analysis and meta-regression, the disparities in sample size between the subgroups limited some of the conclusions. Additionally, some of our subgroup analysis results exhibited publication bias, which may have compromised the validity of the reported effects.

## 5 Conclusions

Taken together, PE had a significant positive effect on the TAS level, and a significant negative effect on the MDA level, but no effect on the levels of SOD, GPx, and CAT. Further RCTs are required to investigate the optimal PE protocol for people of different ages and BMI.

## Supplementary Material

Supplementary material
