# Supplementary material for: Effects of physical exercise on biomarkers of oxidative stress in healthy subjects: A meta-analysis of randomized controlled trials
Source: Open Life Sci. 2023 Aug 8;18(1):20220668. doi: 10.1515/biol-2022-0668 (PMC10426725; doi:10.1515/biol-2022-0668)
Supplement: Supplementary material [file biol-2022-0668-sm.pdf]

Supplementary material

Supplementary table 1

Example search strategy for the Pubmed database.

((randomized controlled trial [Publication Type])OR (randomized[Title/Abstract]) OR (placebo[Title/Abstract])) AND ((Oxidative Stress[Mesh]) OR (Oxidative Stresses [Title/Abstract]) OR (Stresses, Oxidative[Title/Abstract]) OR (Stress, Oxidative[Title/Abstract]) OR (Reactive Oxygen Species[Mesh]) OR (Oxygen Species, Reactive[Title/Abstract]) OR (Active Oxygen[Title/Abstract]) OR (Oxygen, Active[Title/Abstract]) OR (Oxygen Radicals[Title/Abstract]) OR (Pro-Oxidants[Title/Abstract]) OR (Pro Oxidants[Title/Abstract]) OR (Reactive Nitrogen Species[Mesh]) OR (Species, Reactive Nitrogen[Title/Abstract]) OR (Nitrogen Species, Reactive[Title/Abstract]) OR (Nitric Oxide[Mesh]) OR (Antioxidants[Mesh]) OR (Antioxidant Effect[Title/Abstract]) OR (Effect, Antioxidant[Title/Abstract]) OR (Anti-Oxidant Effects[Title/Abstract]) OR (Anti Oxidant Effects[Title/Abstract]) OR (Effects, Anti-Oxidant[Title/Abstract]) OR (Antioxidant Effects[Title/Abstract]) OR (Effects, Antioxidant [Title/Abstract]) OR (Anti-Oxidant Effect[Title/Abstract]) OR (Anti Oxidant Effect[Title/Abstract]) OR (Effect, Anti-Oxidant [Title/Abstract]))AND ((Exercise[Mesh]) OR (Exercises[Title/Abstract]) OR (Exercise, Physical[Title/Abstract]) OR (Exercises, Physical[Title/Abstract]) OR (Physical Exercise [Title/Abstract]) OR (Physical Exercises[Title/Abstract]) OR (Exercise, Isometric[Title/Abstract]) OR (Exercises, Isometric [Title/Abstract]) OR (Isometric Exercises[Title/Abstract]) OR (Isometric Exercise[Title/Abstract]) OR (Exercise, Aerobic [Title/Abstract]) OR (Aerobic Exercises[Title/Abstract]) OR (Exercises, Aerobic[Title/Abstract]) OR (Aerobic Exercise [Title/Abstract]) OR (Exercise Therapy[Mesh]) OR (Therapy, Exercise[Title/Abstract]) OR (Exercise Therapies[Title/Abstract]) OR (Therapies, Exercise[Title/Abstract]) OR (Exercise Movement Techniques[Mesh]) OR (Movement Techniques, Exercise[Title/Abstract]) OR (Exercise Movement Technics[Title/Abstract]) OR (Pilates-Based Exercises[Title/Abstract]) OR (Exercises, Pilates-Based[Title/Abstract]) OR (Pilates Based Exercises[Title/Abstract]) OR (Pilates Training[Title/Abstract]) OR (Training, Pilates[Title/Abstract]))

Example search strategy for other databases.

(("randomized controlled trial" OR randomized OR placebo) AND ("Oxidative Stress" OR "Reactive Oxygen Species" OR "Active Oxygen" OR "Oxygen, Active" OR "Oxygen Radicals" OR "Pro-Oxidants" OR "Pro Oxidants" OR "Reactive Nitrogen Species" OR "Species, Reactive Nitrogen" OR "Nitrogen Species, Reactive" OR "Nitric Oxide" OR "Antioxidants" OR "Antioxidant Effect" OR "Effect, Antioxidant" OR "Anti-Oxidant Effects" OR "Antioxidant Effects" OR "Anti-Oxidant Effect") AND ("Exercise" OR "Physical Exercise" OR "Exercise Therapy" OR "Exercise Movement Techniques" OR "Pilates-Based Exercises")
